# Patchy Distributions of Competitors Affect the Growth of a Clonal Plant When the Competitor Density Is High

**DOI:** 10.1371/journal.pone.0078221

**Published:** 2013-10-21

**Authors:** Wei Xue, Lin Huang, Bi-Cheng Dong, Ming-Xiang Zhang, Fei-Hai Yu

**Affiliations:** School of Nature Conservation, Beijing Forestry University, Beijing, China; University of Saskatchewan, Canada

## Abstract

Environments are patchy in not only abiotic factors but also biotic ones. Many studies have examined effects of spatial heterogeneity in abiotic factors such as light, water and nutrients on the growth of clonal plants, but few have tested those in biotic factors. We conducted a greenhouse experiment to examine how patchy distributions of competitors affect the growth of a rhizomatous wetland plant *Bolboschoenus planiculmis* and whether such effects depend on the density of the competitors. We grew one ramet of *B. planiculmis* in the center of each of the experimental boxes without competitors (*Schoenoplectus triqueter*), with a homogeneous distribution of the competitors of low or high density, and with a patchy distribution of the competitors of low or high density. The presence of competitors markedly decreased the growth (biomass, number of ramets, number of tubers and rhizome length) of the *B. planiculmis* clones. When the density of the competitors was low, the growth of *B. planiculmis* did not differ significantly between the competitor patches and competitor-free patches. However, when the density of the competitors was high, the growth of *B. planiculmis* was significantly higher in the competitor-free patches than in the competitor patches. Therefore, *B. planiculmis* can respond to patchy distributions of competitors by placing more ramets in competition-free patches when the density of competitors is high, but cannot do so when the density of competitors is low.

## Introduction

Environments are spatially patchy with regard to not only abiotic factors (e.g. light, water and nutrients) but also biotic ones (e.g. competitors) [1]–[5]. Many clonal plants have the ability to form a large network of asexual individuals (ramets) connected through horizontal structures such as stolons, rhizomes and roots, so that they often experience environmental heterogeneity [2], [6], [7]. Numerous studies have examined effects of spatial heterogeneity in abiotic factors such as light, water and nutrients on the growth of clonal plants [3]–[6], [8]–[10]. These studies generally show that clonal plants can benefit from spatial heterogeneity by concentrating ramets, roots and/or shoots in favorable patches [11], [12]. However, relatively few studies have tested the effects of spatial heterogeneity in biotic factors such as conspecific competitors [2], [4], [13]–[16].

In a plant community, the spatial distributions of individuals are in many cases not homogeneous but patchy [17], and the presence of conspecific neighbor plants can compete with the target clonal plant for not only resources (light, water and nutrients) but also physical space [18], [19]. Therefore, a heterogeneous distribution of competitors may create an environment with spatial heterogeneity of both resources and physical space. Since clonal plants often experience such environments in natural communities, they may have developed some strategies to cope with such a type of heterogeneity [6], [7], [12].

Competition is often density-dependent [15]. When the density of competitors is low, competition between clonal plants and their conspecific neighbors may be weak [20], [21]. Under such conditions, the contrast of resources and/or space between the patches with competitors and those without competitors is low [4], [15]. In this case, a patchy distribution of competitors may have little effect on the growth of clonal plants. In contrast, if the density of competitors is high, competition between clonal plants and their conspecific neighbors will be strong [20], [21]. In this case, a patchy distribution of competitors may greatly affect the growth and ramet distribution of clonal plants. However, we know little about how effects of the patchy distribution of competitors on clonal plants depend on the density of competitors.

In a greenhouse experiment, we grew the rhizomatous wetland plant *Bolboschoenus planiculmis* in three homogeneous treatments either without competitors (*Schoenoplectus triqueter*) or with a homogeneous distribution of the competitors of low or high density, and two heterogeneous treatments with a patchy distribution of the competitors of low or high density. Specifically, we addressed (1) whether heterogeneous distributions of competitors affect the biomass and ramet distribution of *B. planiculmis* in different patches and, (2) if so, whether such effects depend on the density of competitors.

## Materials and Methods

### The species

*Bolboschoenus planiculmis* (F. Schmidt) T. V. Egorova (Cyperaceae), previously called *Scirpus planiculmis* F. Schmidt, is a perennial rhizomatous herb and commonly inhabits wetlands [22]. It is distributed in China, Central Europe, Russia, the Far East, Central Asia, Japan and Iran [22]. Its rhizomes can branch intensively and form tubers. Aboveground shoots come out in early spring and die in winter, whereas belowground tubers can overwinter and sprout to form new shoots [23]. The shoots can grow to a height of 0.6 to 1.0 m [22]. The density of this species ranges from 20 to 350 ramets per m^2^ in the field. It is widely distributed in wetland habitats such as lakes, riverbanks and swamps [24].

The competitor species, *Schoenoplectus triqueter* (Linnaeus) Palla, previously called *Scirpus triqueter* L., is a rhizomatous perennial herb [25]. It produces trigonous stems of about 100 cm tall [26]. This species is widely distributed in the wetland all over the world, and frequently co-occurs and compete with *B. planiculmis* [27], [28]. The density of this species ranges from 30 to 400 ramets per m^2^ in the field (personal observation). It is commonly distributed in wet places such as river banks, ditches, ponds and swamps [24].

### Sampling and cultivation

On 15 April 2012, we collected more than 100 ramets of *B. planiculmis* and 1000 ramets of *S. triqueter* from the bank of the Beisha River in Beijing (40.134°N, 116.329°E). The soil where the ramets were collected is mainly sand with little clay. Collections of the ramets do not need a special permit from any local authorities. Each ramet was planted into a small pot (10 cm in diameter) in a greenhouse at Forest Science Co. Ltd. of Beijing Forestry University in Beijing. On 30 April 2012, after 15 days of recovery, we selected 30 similar-sized ramets of *B. planiculmis* and 468 similar-sized ramets of *S. triqueter* for the use of this experiment. Initial biomass of *B. planiculmis* was 0.182 ± 0.040 g (mean ± SE, n = 12) and initial height was 27.5 ± 1.7 cm.

### Experimental design

**Figure 1:**
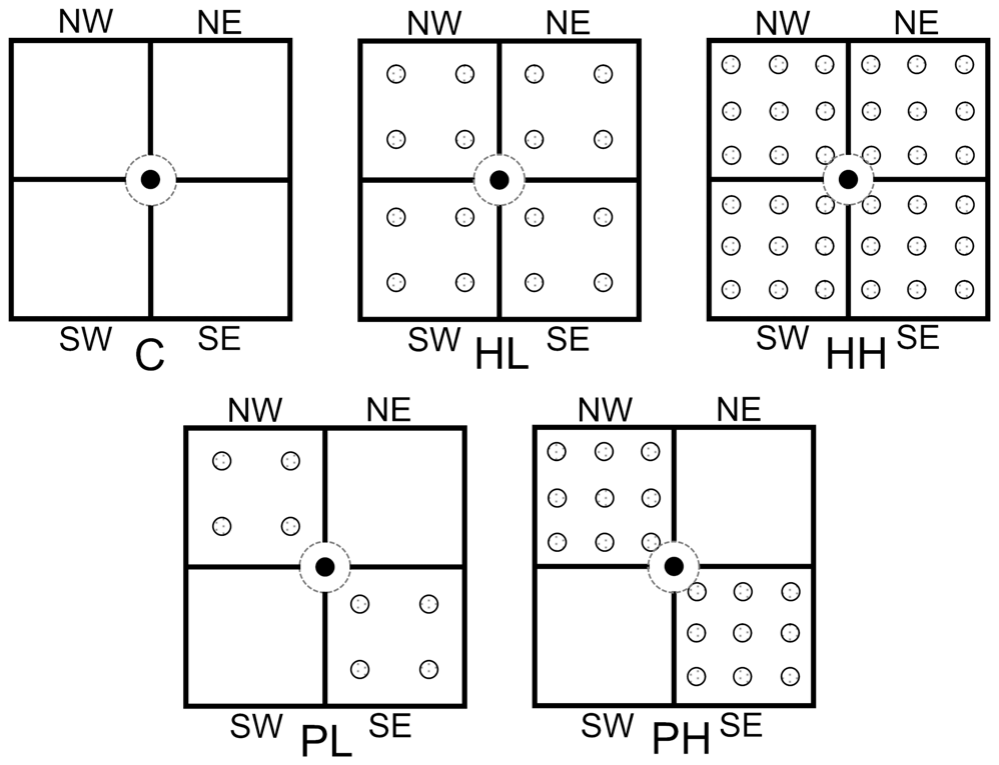
Schematic representation of the experimental design. The experiment consisted of three homogeneous treatments with no competitors (C), a homogeneous distribution of competitors at low density (HL) or high density (HH) and two heterogeneous treatments with a patchy distribution of competitors at low density (PL) or high density (PH). Each box was divided equally into four patches, with two patches in the direction of northeast to southwest (NE-SW) and two patches in the direction of northwest to southeast (NW-SE). For the two heterogeneous treatments, the competitors were distributed in NW-SE patches. Black dots and shaded dots mark the positions where the target plant of *Bolboschoenus planiculmis* and the competing plants of *Schoenoplectus triqueter* were initially grown.

The experiment used a randomized, complete block design and had three homogeneous and two heterogeneous (patchy) treatments (Fig. 1). The three homogeneous treatments were without the competitor *S. triqueter* (coded as “C”, i.e. the control) or with a homogeneous distribution of the competitors of low density (HL) or high density (HH). The two heterogeneous treatments were a patchy distribution of the competitors of low density (PL) or high density (PH). There were six replicate boxes in each treatment, making a total of 30 experimental boxes.

We divided each box into four equal patches (13 cm × 13 cm), with two patches distributed in the direction of northeast to southwest (NE-SW) and two in the direction of northwest to southeast (NW-SE; Fig. 1). We stuck four plastic boards to the sides and bottom of each box to reduce the chance of the *S. triqueter* ramets in plant (competitor) patches to spread into adjacent open patches (no *S. triqueter*), but left a 4.3-cm-diameter circular section at the center free of board so that *B. planiculmis* ramets and rhizomes could grow into each of the four patches (Fig. 1). Each box was then filled up with a 1:1 (v:v) mixture of river sand and peat (Pindstrup Seeding; Pindstrup Mosebrug A/S, Pindstrup, Denmark) as well as 8.8 g slow-release fertilizer (15N-11P-13K-2Mg; Osmocote 301, Scotts, Marysville, Ohio, USA).

The 30 ramets of the target species *B. planiculmis* were then planted at the center of the 30 boxes (26 cm long × 26 cm wide × 13 cm deep). In C, no ramet of the competitor *S. triqueter* was present in the box (Fig. 1). In HL and HH, 16 and 36 ramets of *S. triqueter* were planted homogeneously in a box, respectively (Fig. 1). In PL and PH, 8 and 18 ramets of *S. triqueter* were planted in two of the four patches (i.e. NW-SE patches in a box, respectively, Fig. 1).

The experiment was maintained for 90 days (from 30 April to 28 July 2012) in the same greenhouse as for pre-cultivation. During the experiment, the mean temperature and the relative humidity in the greenhouse were 26.5°C and 59.2%. The positions of the boxes were changed systematically every two weeks, and tap water was supplied to all plants regularly once the surface of the soil became dry.

### Harvest and measurement

At the end of the experiment, all ramets of *B. planiculmis* were harvested and parent ramets and offspring ramets were harvested separately. For all the five treatments, we harvested the new ramets in the patches of northeast to southwest (NE-SW) and in the patches of northwest to southeast (NW-SE) separately. Thus, in the two patchy treatments (PL and PH) ramets of *B. planiculmis* distributed in the same type of patches were combined and in three homogeneous treatments (C, HL and HH) ramets in the opposite two patches were combined. We counted number of ramets and number of tubers and measured rhizome length. Then, all plants were divided into roots, stems, laminae, tubers and rhizomes. Dry mass of all plant parts was determined after oven-dried at 70°C for at least 48 h. At harvest, there were no ramets of *S. triqueter* in the open patches.

### Data analysis

Before analysis, values of biomass of *B. planiculmis* were transformed to square root to increase normality and homogeneity of variance. At the whole plant (box) level, we used one-way ANOVA followed by Duncan’s test to compare the differences in the growth (total biomass, lamina mass, root mass, stem mass, rhizome mass and tuber mass, number of ramets, number of tubers and rhizome length) of *B. planiculmis* among the five treatments. At the patch level, we used one-way ANOVA followed by Duncan’s tests to compare the differences in the growth in the NE-SW patches and in the NW-SE patches separately among the five treatments. We used one-way ANOVA followed by linear contrasts to compare the differences between the growth measures of *B. Planiculmis* in the NE-SW patches and the NW-SE patches in each treatment [13]. All analyses were performed with SPSS 17.0 (SPSS, Chicago, IL, USA). The difference was considered significant if *P*<0.05.

## Results

### Effects at the whole plant (box) level

**Figure 2:**
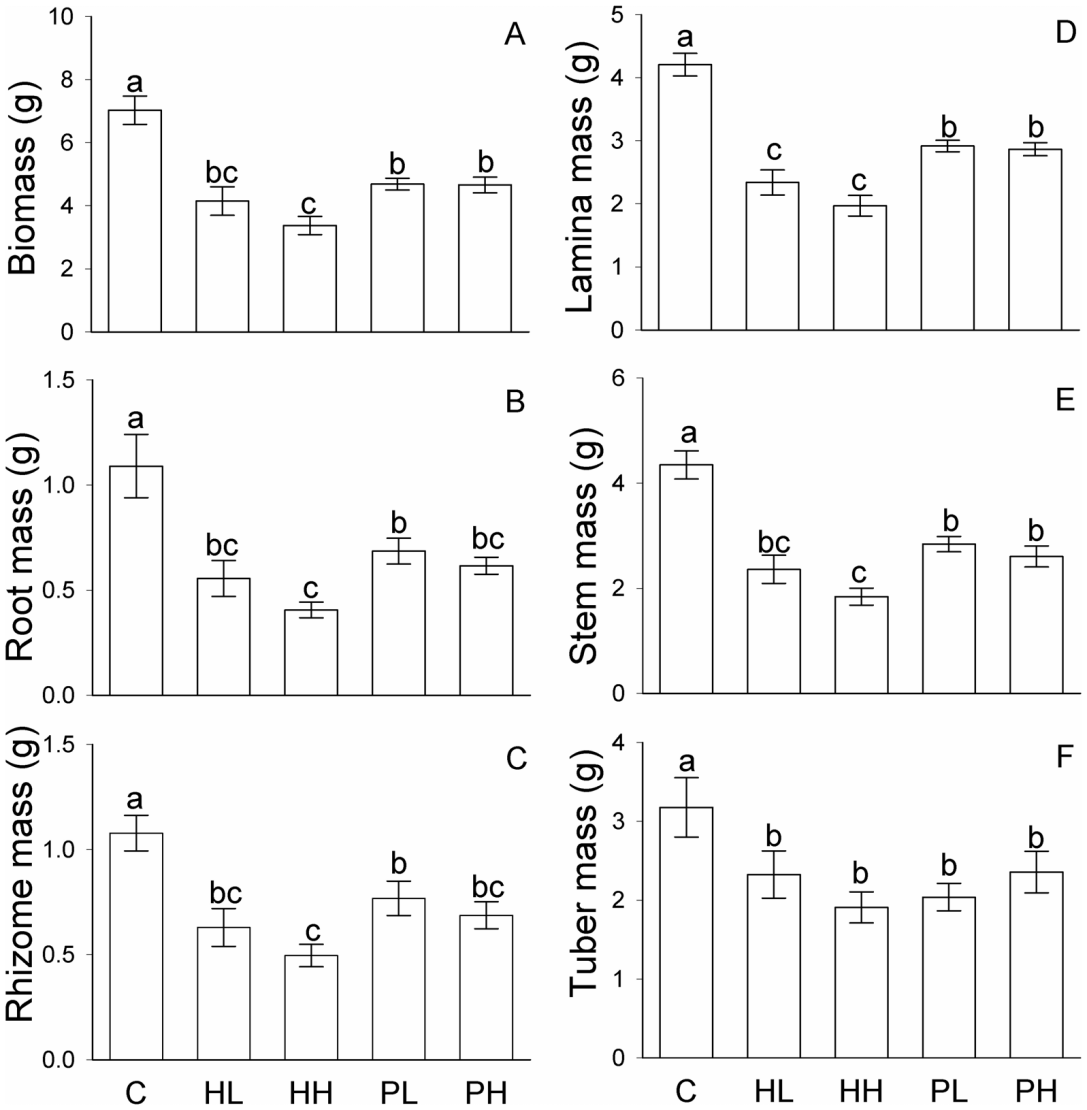
Biomass of *Bolboschoenus planiculmis* at the whole plant (box) level under the five treatments. Mean values (± 1 SE) are given. C, HL, HH, PL and PH represent the homogeneous treatment with no competitors, homogeneous treatment with competitors of low density, homogeneous treatment with competitors of high density, patchy treatment with competitors of low density, and patchy treatment with competitors of high density, respectively. Bars sharing the same letters are not different at *P* = 0.05.

**Figure 3:**
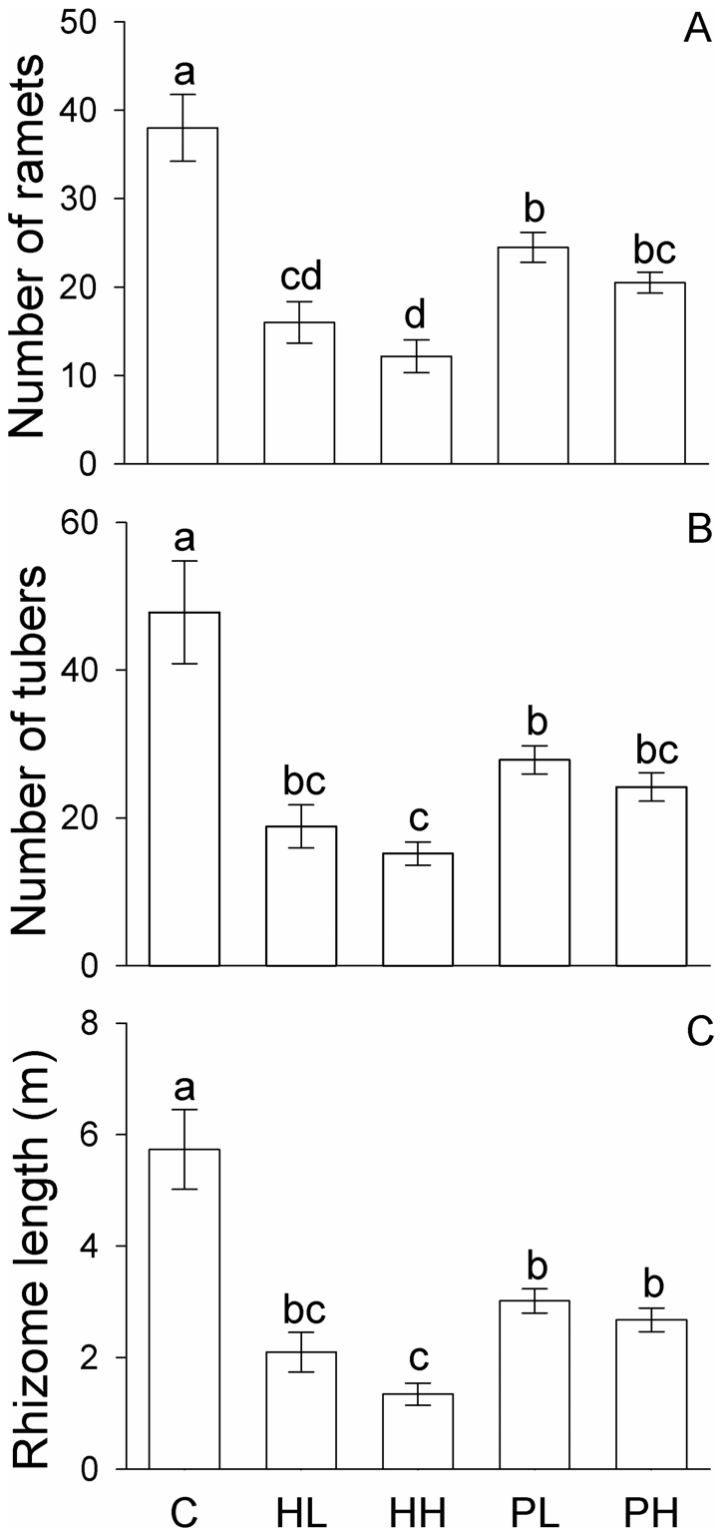
Clonal growth measures of *Bolboschoenus planiculmis* at the whole plant (box) level under the five treatments. Mean values (± 1 SE) are given. C, HL, HH, PL and PH represent the homogeneous treatment with no competitors, homogeneous treatment with competitors of low density, homogeneous treatment with competitors of high density, patchy treatment with competitors of low density, and patchy treatment with competitors of high density, respectively. Bars sharing the same letters are not different at *P* = 0.05.

In homogeneous conditions, compared with the control (C), the presence of competitors (*S. triqueter*) significantly decreased all growth measures (total biomass, root mass, rhizome mass, lamina mass, stem mass, tuber mass, number of ramets, number of tubers and rhizome length) of *B. planiculmis* (*F*_4_, _29_ = 3.30 – 30.38, *P* = 0.000 – 0.027, Table 1; Figs. 2 and 3). However, none of the growth measures differed significantly between the homogeneous, low density (HL) and the homogeneous, high density treatment (HH; Figs. 2 and 3). All growth measures in the two patchy treatments (PL and PH) were significantly smaller than those in the control (Figs. 2 and 3), and total biomass, lamina mass, stem mass, number of ramet and rhizome length in PL and PH were also greater than those in HH (Figs. 2A, D, E and 3A, C).

**Table 1:** Effects of the treatments on the growth of *Bolboschoenus planiculmis* at the whole plant (box) level.

	*F*_4, 29_	*P*
Biomass	15.86	<0.001
Root mass	8.93	<0.001
Rhizome mass	8.19	<0.001
Lamina mass	30.38	<0.001
Stem mass	19.22	<0.001
Tuber mass	3.30	0.027
No. of ramets	18.07	<0.001
No. of tubers	12.21	<0.001
Rhizome length	18.10	<0.001

*F*-values, degree of freedoms and *P*-values are based on one-way ANOVA.

### Effects at the patch level

**Figure 4:**
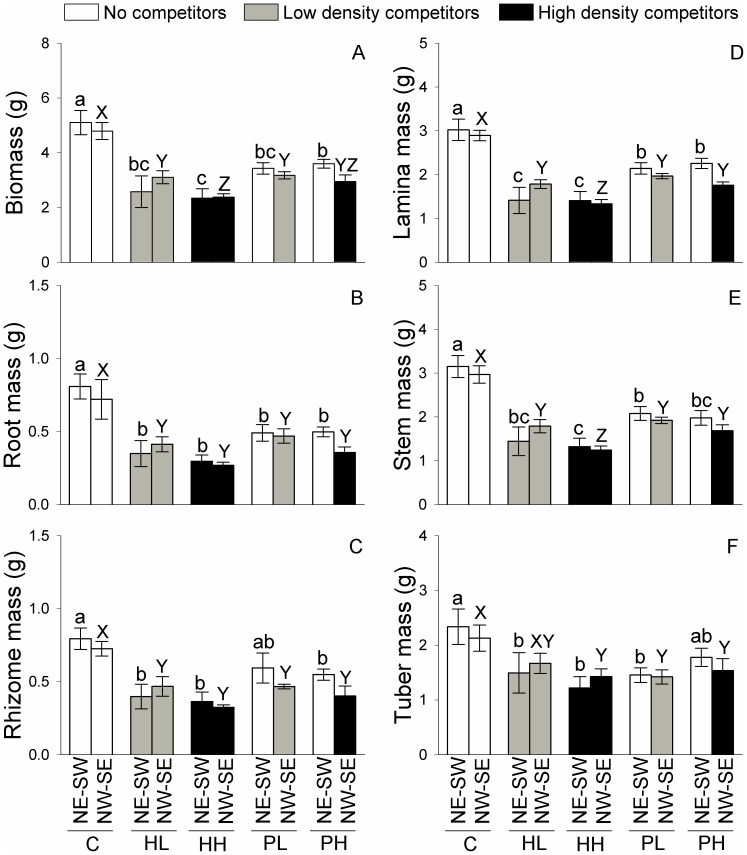
Biomass of *Bolboschoenus planiculmis* in the patches of northeast-southwest (NE-SW) and northwest-southeast (NW-SE) under the five treatments. Mean values (± 1 SE) are given. C, HL, HH, PL and PH represent the homogeneous treatment with no competitors, homogeneous treatment with competitors of low density, homogeneous treatment with competitors of high density, patchy treatment with competitors of low density, and patchy treatment with competitors of high density, respectively. For growth measures in NE-SW patches bars sharing the same small letters (a-c) are not different at *P* = 0.05, and for those in NW-SE patches bars sharing the same capital letters (X-Z) are not different at *P* = 0.05.

**Figure 5:**
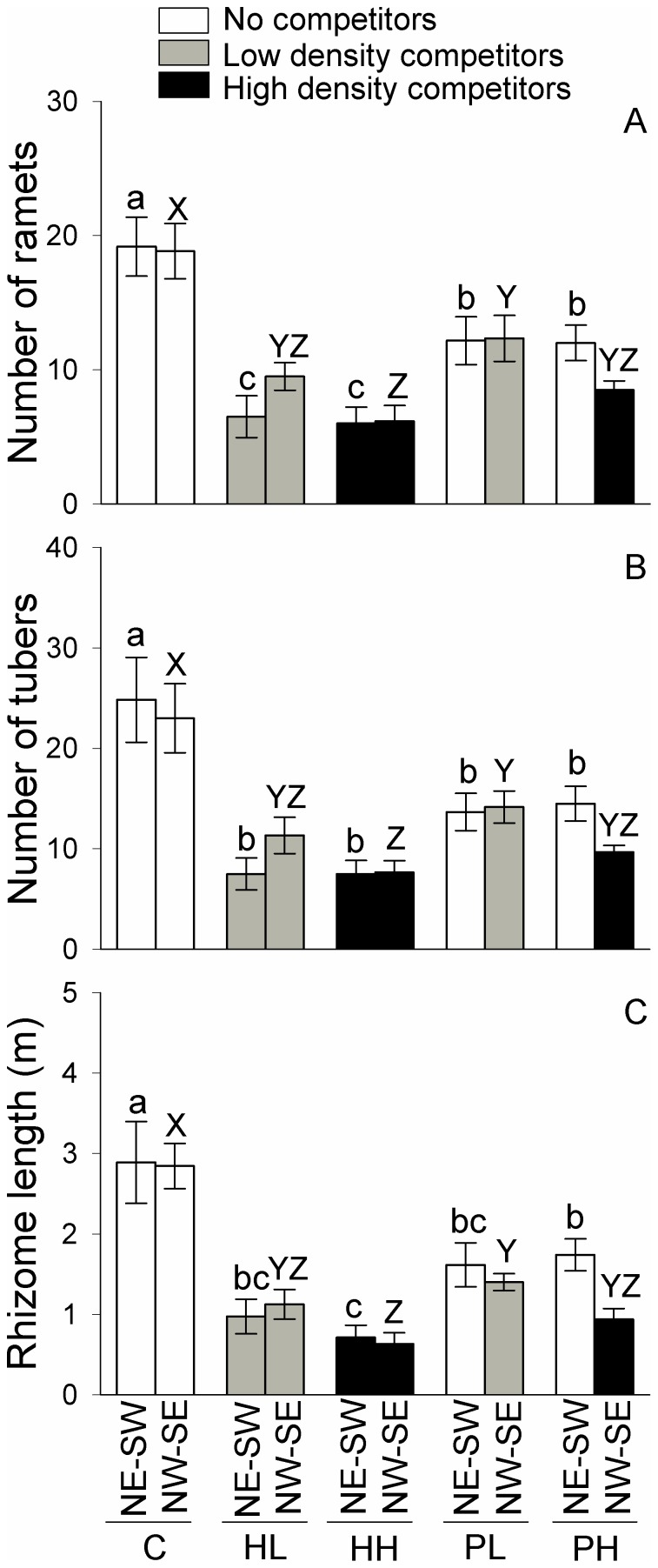
Clonal gowth measures of *Bolboschoenus planiculmis* in the patches of northeast-southwest (NE-SW) and northwest-southeast (NW-SE) under the five treatments. Mean values (± 1 SE) are given. C, HL, HH, PL and PH represent the homogeneous treatment with no competitors, homogeneous treatment with competitors of low density, homogeneous treatment with competitors of high density, patchy treatment with competitors of low density, and patchy treatment with competitors of high density, respectively. For growth measures in NE-SW patches bars sharing the same small letters (a-c) are not different at *P* = 0.05, and for those in NW-SE patches bars sharing the same capital letters (X-Z) are not different at *P* = 0.05.

None of the growth measures differed between the NE-SW and the NW-SE patches in the three homogeneous treatments (C, HL and HH) or the patchy, low density treatment (PL; *F*_1, 10_ = 0.01 – 2.57, *P* = 0.140 – 0.948, Table 2; Figs. 4 and 5). In the patchy, high density treatment (PH), however, total biomass, root mass, lamina mass, number of ramets, number of tubers and total rhizome length were all significantly higher in the NE-SW patches (without competitors) than in the NW-SE patches (with competitors; *F*_1, 10_ = 5.09 – 12.81, *P* = 0.005 – 0.048, Table 2; Figs. 4 and 5).

**Table 2:** Effects of patch position (NE-SW vs. NW-SE patches) on the growth of *Bolboschoenus planiculmis* under each of the five treatments.

		NE-SW patches vs. NW-SE patches				
		C	HL	HH	PL	PH
Biomass	*F*_1, 10_	0.33	0.71	0.01	1.09	5.09
	*P*	0.577	0.421	0.930	0.321	0.048
Root mass	*F*_1, 10_	0.30	0.38	0.35	0.08	7.81
	*P*	0.594	0.551	0.568	0.777	0.019
Rhizome mass	*F*_1, 10_	0.60	0.41	0.37	1.51	3.52
	*P*	0.456	0.539	0.557	0.247	0.090
Lamina mass	*F*_1, 10_	0.23	1.37	0.11	1.50	12.81
	*P*	0.645	0.269	0.751	0.249	0.005
Stem mass	*F*_1, 10_	0.32	0.94	0.13	0.82	1.84
	*P*	0.585	0.355	0.726	0.387	0.205
Tuber mass	*F*_1, 10_	0.27	0.18	0.67	0.03	0.76
	*P*	0.616	0.680	0.431	0.864	0.403
No. of ramets	*F*_1, 10_	0.01	2.57	0.01	0.01	5.61
	*P*	0.914	0.140	0.923	0.948	0.039
No. of tubers	*F*_1, 10_	0.11	2.55	0.01	0.04	6.82
	*P*	0.743	0.141	0.926	0.842	0.026
Rhizome length	*F*_1, 10_	0.01	0.28	0.15	0.54	11.24
	*P*	0.940	0.606	0.704	0.481	0.007

*F*-values, degree of freedoms and *P*-values are based on linear contrasts between the NE-SW and NW-SE patches. C, HL, HH, PL and PH represent the homogeneous treatment with no competitors, homogeneous treatment with competitors of low density, homogeneous treatment with competitors of high density, patchy treatment with competitors of low density, and patchy treatment with competitors of high density, respectively.

All growth measures except rhizome mass and tuber mass in the NE-SW patches (no competitors) were markedly greater in the control (C) than in the patchy, low density (PL) and the patchy, high density treatment (PH; Figs. 4 and 5). Rhizome mass in the NE-SW patches was greater in C than in PH, but did not differ between C and PL (Fig. 4C). Tuber mass in the NE-SW patches was greater in C than in PL, but did not differ between C and PH (Fig. 4F).

None of the growth measures in the NW-SE patches differed significantly between the homogenous, low density and patchy, low density treatments (in the low density patches of HL *vs.* PL; Figs. 4 and 5). In the NW-SE patches, none of the growth measures except lamina mass and stem mass differed significantly between the homogeneous, high density and patchy, high density treatments (in the high density patches of HH *vs.* PH; Figs. 4 and 5).

## Discussion

Not surprisingly, the presence of competitors (*S. triqueter*) markedly decreased the growth of *B. planiculmis*, agreeing with previous findings on other clonal plants [29]-[35]. The presence of competitors, especially in a high density, may greatly decrease not only local resources (light and nutrients) but also physical space available for the growth of the *B. planiculmis* ramets [36], [37]. This is because dense shoots of competitors could block the spread of the aboveground shoots of *B. planiculmis* and dense roots and rhizomes of competitors could obstruct the spread of the rhizomes (clonal growth) of *B. planiculmis*. In the present study, competition for water was less likely because water was added once the soil surface was dry. Therefore, the negative effects of the competitors on *B. planiculmi*on were most likely due to competition for light, nutrients and physical space, and patchy distribution of competitors might create an environment with not only a patchy distribution of resources but also a patchy distribution of available physical space [13], [38]–[40].

When growing in the environment with a patchy distribution of competitors, *B. planiculmis* produced markedly more biomass, ramets and potential ramets (tubers) in the competitor-free (NE-SW) patches than in the competitor (NW-SE) patches when the density of the competitors was high. These results suggest that in a heterogeneous environment with a high density of competitors *B. planiculmis* could reduce the chance to grow into competitor patches (with less resources and physical space) and increase the chance to spread in competitor-free patches (with abundant resources and space) [2], [4], [13], [15]. These responses are thought to be adaptive because they potentially increase the chance of the whole genet to survive and to spread [41]–[45].

When the density of the competitors was low, there was no significant difference in biomass and ramet production between the competitor patches and competitor-free patches. On the other hand, compared to the control (no competitors) biomass and ramet production were greatly reduced in the homogeneous, low density treatment. One likely explanation is that a relatively large amount of resources and physical space were still available in the competitor patches in the heterogeneous, low density treatment compared to those in the heterogeneous, high density treatment [20], [21]. In the heterogeneous, low density treatment, therefore, rhizomes and ramets of *B. planiculmis* could grow into the competitor patches with the help of the resources translocated from the parent ramet and also the ramets located in the competitor-free patches [12], [46]–[47]. Meanwhile, resource translocation to the ramets of *B. planiculmis* in the competitor patches greatly reduced the growth of the ramets growing in the competitor-free patches.Consequently, we could not find a significant difference between the competitor patches and competitor-free patches.

The growth and clonal reproduction of *B. planiculmis* in the NE-SW (competitor-free) patches were larger in the control than in the patchy, low density and patchy, high density treatments, suggesting that connections to the ramets of *B. planiculmis* in the competitor patches brought great costs to the ramets in the competitor-free patches [46], [48]–[50]. This result further indicates that translocation of resources (physiological integration) took place from the ramets in the competitor-free patches to the ramets in the competitor patches though rhizomes [51]–[53]. Such resource translocation significantly increased lamina mass and stem mass and tended to increase total biomass and clonal production of the ramets growing in the competitor patches in the patchy, high density treatment [54]–[55].

We conclude that spatial heterogeneity in the distribution of conspecific competitors can greatly affect the growth of clonal plants when the density of the competitors is high [15], [38]. Clonal plants can respond to patchy distribution of competitors by positioning more biomass, ramets and potential ramets (tubers) in open (competition-free) patches. Because in many ecosystems conspecific plants are often distributed in patches [17], such responses may confer clonal plants with advantages and thus may affect the structure of plant communities and contribute to species co-existence [17], [18], [56], [57].
